# Joint pain and osteoarthritis in former recreational and elite cricketers

**DOI:** 10.1186/s12891-019-2956-7

**Published:** 2019-12-12

**Authors:** He Cai, Garrett S. Bullock, Maria T. Sanchez-Santos, Nicholas Peirce, Nigel K. Arden, Stephanie R. Filbay

**Affiliations:** 1 West China Hospital of Stomatology, Sichuan University, No. 14, Section 3, South Renmin Road, Chengdu, 610041 China; 2 Centre for Sport, Exercise and Osteoarthritis Research Versus Arthritis, Nuffield Department of Orthopaedics, Rheumatology and Musculoskeletal Sciences, University of Oxford, Windmill Road, Oxford, OX3 7LD UK; 3 Centre for Statistics in Medicine & Rehabilitation Research in Oxford, Nuffield Department of Orthopaedics, Rheumatology and Musculoskeletal Sciences, University of Oxford, Windmill Road, Oxford, OX3 7LD UK; 4 England and Wales Cricket Board, Lords Cricket Ground, St John’s Wood Road, London, NW8 8QZ UK; 5 National Centre for Sports and Exercise Medicine and National Cricket Performance Centre, Loughborough University, Loughborough, LE11 3TU UK

**Keywords:** Cricket, Retired athletes, Recreational sport, Knee, Spine, Hand, Shoulder, Hip, Ankle

## Abstract

**Background:**

Sport participants are at increased risk of joint pain and osteoarthritis. A better understanding of factors associated with joint pain and osteoarthritis in this population could inform the development of strategies to optimise their long-term joint health. The purpose of the study was to describe the prevalence of joint pain and osteoarthritis in former cricketers, and determine whether playing position, playing standard (i.e. elite or recreational standard) and length-of-play are associated with region-specific joint pain.

**Methods:**

The data were from the Cricket Health and Wellbeing Study (CHWS), a cohort of 2294 current and former cricketers (played ≥1 season) in England and Wales. For this study, eligible individuals had to be aged ≥30 years and be a former cricket participant. Joint pain was defined as region-specific (hip/knee/ankle/shoulder/hand/back) pain on most days of the last month. Osteoarthritis was defined as joint-specific doctor-diagnosed osteoarthritis. Logistic regression was used to calculate unadjusted and adjusted (for history of joint injury resulting in > 4 weeks of reduced activity +/− age) odds ratios (ORs) and 95% confidence intervals (95% CIs).

**Results:**

846 individuals from the CHWS were former cricketers aged ≥30 years (3% female, aged median 62(IQR 54–69) years, 62% played cricket recreationally, median 33(IQR 21–41) cricket seasons). One-in-two (48%) reported joint pain and 38% had been diagnosed with osteoarthritis. Joint pain and OA were most common in the knee (23% pain, 22% osteoarthritis), followed by the back (14% pain, 10% osteoarthritis) and hand (12% pain, 6% osteoarthritis). After adjusting for injury, bowlers had greater odds of shoulder pain (OR (95% CI) 3.1(1.3, 7.4)) and back pain (3.6(1.8, 7.4)), and all-rounders had greater odds of knee (1.7(1.0, 2.7)) and back pain (2.1(1.0, 4.2)), compared to batters. Former elite cricketers had greater odds of hand pain (1.6(1.0, 2.5)) than former recreational cricketers. Playing standard was not related to pain at other sites, and length-of-play was not associated with joint pain in former cricketers.

**Conclusions:**

Every second former cricketer experienced joint pain on most days of the last month, and more than one in three had been diagnosed with osteoarthritis. Compared with batters, bowlers had higher odds of shoulder and back pain and all-rounders had higher odds of back and knee pain. Elite cricket participation was only related to higher odds of hand pain compared with recreational cricket participation.

## Background

Sport participation has a positive impact on quality of life (QOL) and mental health [1]. However, sport participation also has a high injury incidence, which predisposes sport participants to osteoarthritis (OA) [2, 3]. Musculoskeletal disorders, of which OA is the most common, directly and indirectly costs the United States, Canada, United Kingdom, and France 1–2.5% of their gross national product each year [4]. The substantial burden of OA is related to chronic pain (the most common symptom of OA), impaired function, and reduced QOL [5, 6]. Due to the high prevalence of OA in former sport participants and the negative personal and societal impacts of OA, understanding factors related to OA and joint pain in former sport participants is of great importance. Such information has potential to inform strategies to optimize long-term joint health for sport participants.

Cricket is popular throughout the world with 104 countries or geographical areas registered in the International Cricket Council [7]. However, cricket has a high injury rate and associated risk of OA development [2, 8, 9]. Cricket injuries have been reported to be as high as 53 injuries per 10,000 athlete exposures, with injury prevalence differing per playing position (fast bowlers: 21%; other positions: 5–7%) [2]. Different injury rates based on playing position may be related to contrasting psychological and biomechanical demands [10], which may result in different risk of OA development. However, it is unknown if the location or prevalence of joint pain or OA in former cricketers differs according to playing position, after accounting for prevalence of cricket-related joint injury. Such information may have important implications for OA prevention strategies and may be used to inform cricketers about the long-term musculoskeletal risks associated with specific playing positions.

As many as 44% of former professional cricketers develop OA [9]. However, there were nearly 1.7 million British people playing cricket recreationally in 2013 [11]. Hence, it is also important to take into account the large number of recreational cricketers. The rates of joint pain and OA in former non-professional cricketers has not been investigated and it is not clear how musculoskeletal health compares between former cricketers of different playing standards. Furthermore, it is not clear if playing cricket for a greater number of seasons is associated with worse joint health, after accounting for age and injury.

Using data from the Cricket Health and Wellbeing Study, the purpose of this study was to: i) describe the prevalence of joint pain and OA (in the hip/groin, knee, ankle, shoulder, hand, back), and ii) determine whether predominate playing-position, playing standard and length of play are associated with joint pain (after accounting for injury) in former cricketers aged ≥30 years.

## Methods

**Fig. 1:**
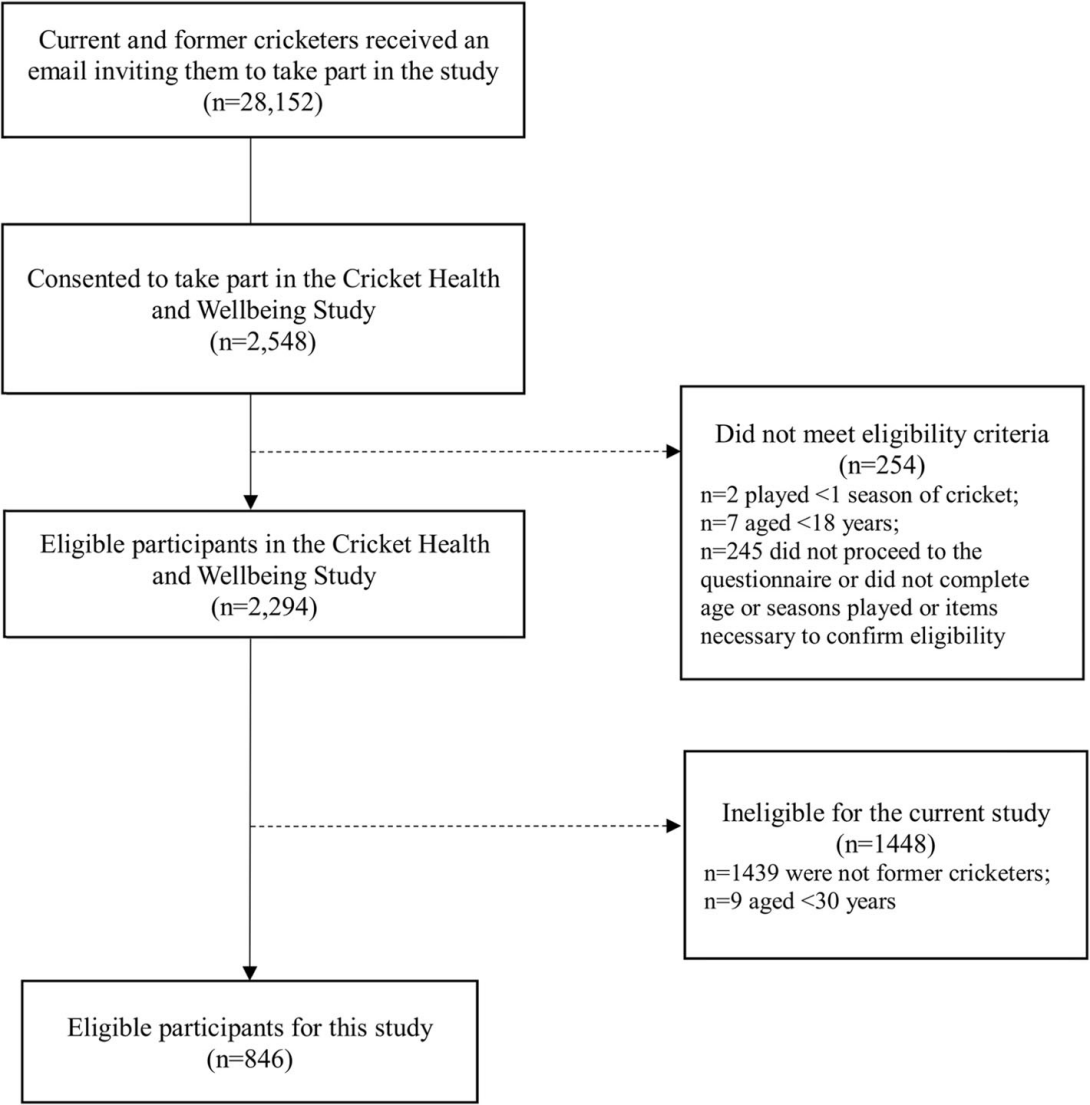
Participant recruitment flow chart

The Cricket Health and Wellbeing Study comprises a large sample of current and former cricket participants in England and Wales, of all playing standards. In March 2017, 28,152 current and former cricketers registered on an online database managed by the England and Wales Cricket Board, who had agreed to be contacted for cricket-related research, received one email containing study information, eligibility criteria, and an electronic link to the consent form and a cross-sectional questionnaire. The eligibility criteria for the Cricket Health and Wellbeing Study were: age ≥ 18 years and had played ≥1 season of cricket. Current and former cricket participants who had played any standard of organised cricket were included. 2548 individuals (9.1% of individuals who received an email invitation) provided written informed consent and 2294 of those were eligible. All participants were recruited over an 8-week period (from 13 March 2017 to 14 May 2017). To be eligible for the current cross-sectional study, individuals were required to be i) former cricketers (no longer playing cricket and did not plan to return to cricket); and ii) aged ≥30 years at the time of questionnaire completion (since OA is rare in people aged < 30 years). Full details of participant recruitment is presented in Fig. 1.

The Cricket Health and Wellbeing Study collected information regarding cricket playing history, cricket-related injury, joint pain and OA, general medical history, physical activity participation, and QOL. The questionnaire was designed to evaluate these outcomes and explain variation in these outcomes using participant characteristics and a variety of cricket-related factors. The questionnaire was developed with involvement from cricketers and in collaboration with the England and Wales Cricket Board. The questionnaire was piloted on 2 current and 2 former cricketers, giving rise to slight revisions of wording to some items (e.g. the response options for playing standard were modified). The questionnaire was developed and managed using RedCap® (Research Electronic Data Capture) software (a web-based data collection application) by an experienced database manager, and tested by three researchers [12]. The RedCap® software enabled the appropriate utilization of branching logic and gave people the option of saving their progress online, and completing the questionnaire at a later time [12].

### Outcomes, explanatory variables, and confounders

#### Outcomes

Joint pain was evaluated using the following questions, ‘*Do you currently experience pain, discomfort, or have any problems in any of your joints?*’ If yes, participants were then asked, ‘*Have you had pain in your hip/groin, knee, ankle, shoulder, hand/finger, spine/back, or other joint(s) on most days of the last month*?’ For the purposes of this study, joint pain was defined as pain on most days of the last month, which was in line with the National Health and Nutrition Examination Survey criteria for joint pain [13] that has been used previously to represent clinical signs of symptomatic OA [14–16]. Osteoarthritis was assessed by asking participants, ‘*Have you ever been told by a doctor that you have osteoarthritis (wear and tear or joint degeneration)?*’ If yes, participants were asked to indicate which joint(s) (hip/groin, knee, ankle, shoulder, hand/finger, spine/back, or other joint(s)).

#### Explanatory variables

To evaluate predominate playing position, participants were asked ‘*What is/was your predominant position(s) of play?*’ Participants could select one or more of the following options: ‘Batting’, ‘Bowling’, ‘All-rounder’, ‘Wicketkeeper’, and ‘Don’t know’. Predominate playing position responses were re-coded into 4 mutually exclusive categories: ‘batter’ (only ‘Batter’ selected); ‘bowler’ (only ‘Bowler’ selected); ‘all-rounder’ (‘All-rounder’ and/or both ‘Batter’ and ‘Bowler’ selected); and ‘wicketkeeper-batter’ (only ‘Wicketkeeper’ or both ‘Wicketkeeper’ and ‘Batter’ selected). For the purposes of multivariable analysis, participants who selected rare combinations of playing positions (e.g. ‘Wicketkeeper’ and ‘Bowler’) were excluded from analysis (*n* = 34). Playing standard was evaluated by asking participants ‘*What was the highest standard of cricket that you played for at least one season?*’ with the following response options: ‘International’; ‘County or premier league’; ‘Academy or county age group’; ‘University’; ‘School’; ‘Village or social’; ‘Don’t know’). Playing standard was dichotomised into ‘recreational standard’ (‘University’, ‘School’, or ‘Village or social’) vs. ‘elite standard’ (‘International’, ‘County or premier league’, or ‘Academy or county age group’). Length of play was assessed by asking participants to respond to ‘*Approximately how many seasons have you played cricket for?*’ with a numeric text response. Due to the skewed distribution, length of play was re-coded into 4-season intervals (i.e. ‘1’ = 1–4 seasons, ‘2’ = 5–8 seasons, ‘10’ = 37–40 seasons etc.). All ‘Don’t know’ responses were excluded from analyses.

#### Confounders

Confounders identified through review of the literature and clinical reasoning included cricket-related region-specific injury and age. Cricket-related injury was evaluated using the following question: ‘*Have you ever had any cricket-related injuries leading to more than 4 weeks of reduced participation in exercise, training or sport*?’ ‘If yes, indicate where (hip/groin, knee, ankle, shoulder, hand/finger, spine/back, other joint(s))’.

### Statistical analysis

Characteristics for former cricketers were described using median and interquartile range (IQR) for continuous variables, and relative and absolute values for categorical variables. A series of binary logistic regressions were performed to assess the relationship between explanatory variables (predominate playing position, playing standard, and length of play) and region-specific pain. Prior to analyses, all underlying logistic regression assumptions were evaluated and met [17, 18]. Crude and adjusted (estimates were adjusted for injury for all analyses, and age in relation to length of play) odds ratios (ORs) and 95% confidence intervals (95% CIs) were estimated. All analyses were conducted in IBM SPSS Statistics 21 (SPSS Inc., IBM, Chicago, Illinois). Where clinical reasoning or literature suggested possible interactions between variables, interaction terms were assessed between explanatory variables and confounders [19, 20]. We planned to include interactions in regression models if their effects on the outcome were statistically significant; however none of the interaction terms investigated had a significant effect on the outcomes. Due to the small amount of missing data (joint pain: 1%; OA: 1%; cricket-related injury: 2%; age: 0%; playing position: 2%; playing standard: 2%; and length of play: 1%), complete-case analyses were performed.

## Results

The 846 former cricketers that participated in this study, were aged median 62(IQR 54–69) years; 3% were female; body mass index (BMI): 27.94(25.26–30.74) kg/m^2^; years since retirement: 10(3–18) years; and had played cricket for median 33(21–41) seasons (Table 1). 39% (*n* = 311) of participants were predominately all-rounders, 23% (*n* = 180) were bowlers; 23% (*n* = 183) were batters, and 16% (*n* = 124) were wicketkeeper-batters. 38% (*n* = 318) of participants had played cricket at an elite level (international, county, premier league, academy, county age group) and 62% (*n* = 511) had only played recreationally (university, school, village, social). Among former elite cricketers, 53% (*n* = 165) reported joint pain and 45% (*n* = 136) had been diagnosed with OA. In the subgroup of former recreational cricketers, 46% (*n* = 232) reported joint pain and 34% (n = 165) had been diagnosed with OA.

**Table 1:** Participant characteristics

Characteristics	All former cricketers (*n* = 846)	Former cricketers with joint pain (*n* = 404)	Former cricketers without joint pain (*n* = 431)
Age, years	62 (54–69)	63 (55–70)	62 (54–69)
Body mass index, kg/m^2^	27.94 (25.26–30.74)	28.48 (25.83–31.40)	27.54 (24.68–30.09)
Sex			
Female	24 (3%)	13 (3%)	10 (2%)
Male	815 (97%)	387 (97%)	418 (97%)
Other / Don’t wish to disclose	2 (0%)	1 (0%)	1 (0%)
Cricket-related injury	355 (43%)	193 (49%)	160 (38%)
Main playing position			
Bowler	180 (23%)	98 (26%)	81 (20%)
Wicketkeeper-batter	124 (16%)	53 (14%)	71 (18%)
All-rounder	311 (39%)	165 (43%)	140 (35%)
Batter	183 (23%)	66 (17%)	113 (28%)
Playing standard			
Elite standard	318 (38%)	165 (42%)	149 (35%)
Recreational standard	511 (62%)	232 (58%)	274 (65%)
Length of play, seasons	33 (21–41)	35 (25–42)	31 (20–40)
Years since retirement from cricket, years	10 (3–18)	10 (3–18)	8 (3–18)
Retirement reasons			
Due to chronic pain or injury	317 (37%)	221 (55%)	93 (22%)
Personal or family-related reasons	224 (26%)	91 (23%)	125 (29%)
No longer good at it / or no longer enjoyed it	187 (22%)	83 (21%)	101 (23%)
Age	345 (41%)	170 (42%)	171 (40%)
Lack of time or work related commitments	203 (24%)	90 (22%)	111 (26%)
To focus on another sport/ exercise	57 (7%)	21 (5%)	35 (8%)
Didn’t get along with team mates or coach	7 (1%)	3 (1%)	4 (1%)
Other	109 (13%)	44 (11%)	63 (15%)
Smoker			
Yes	50 (6%)	24 (6%)	26 (6%)
No	616 (73%)	277 (69%)	328 (77%)
Ex-smoker	174 (21%)	100 (25%)	74 (17%)
Employment status			
Full-time	346 (41%)	151 (37%)	189 (45%)
Part-time/casual	93 (11%)	41 (10%)	51 (12%)
Student	1 (0%)	1 (0%)	0 (0%)
Stay at home parent/carer	8 (1%)	5 (1%)	3 (1%)
Retired	385 (46%)	204 (50%)	177 (42%)
Unempleyed	6 (1%)	2 (0%)	4 (1%)
Education			
GCSE/O level/A level	241 (29%)	114 (29%)	124 (29%)
Further/Higher education	551 (66%)	257 (65%)	287 (67%)
Other	45 (5%)	27 (7%)	17 (4%)

• All values are presented as count (proportion) or median (interquartile range);

• All the available data for the participant demographics were reported with the missing data in each variable excluded, for instance, 11 participants with missing joint-pain data were excluded from the subgroups of former cricketers with and without joint pain;

• ‘Joint pain’: ‘*Have you had pain in your hip/groin, knee, ankle, shoulder, hand/finger, spine/back, or other joint(s) on most days of the last month?*’;

• ‘Main playing position’: ‘*What is/was your predominant position(s) of play?*’ Multiple positions could be selected so this was recoded into mutually exclusive categories: ‘batter’ (only ‘Batter’ selected); ‘bowler’ (only ‘Bowler’ selected); ‘all-rounder’ (‘All-rounder’ and/or both ‘Batter’ and ‘Bowler’ were selected); ‘wicketkeeper-batter’ (only ‘Wicketkeeper’ or both ‘Wicketkeeper’ and ‘Batter’ were selected); other combinations of playing positions (e.g. ‘Wicketkeeper’ and ‘Bowler’) (*n* = 34) were excluded;

• All values are presented as count (proportion) or median (interquartile range);

• ‘Playing standard’: ‘*What was the highest standard of cricket that you played for at least one season?*’ Responses were dichotomised into ‘elite standard’ (‘International’, ‘County or premier league’, or ‘Academy or county age group’) and ‘recreational standard’ (‘University’, ‘School’, or ‘Village or social’);

• ‘Length of play’: ‘*Approximately how many seasons have you played cricket for?*’

• ‘Cricket-related injury’: ‘*Have you ever had any cricket-related injuries leading to more than 4 weeks of reduced participation in exercise, training or sport?*’ If yes, indicate where (hip/groin, knee, ankle, shoulder, hand/finger, spine/back, other joint(s)).

**Fig. 2:**
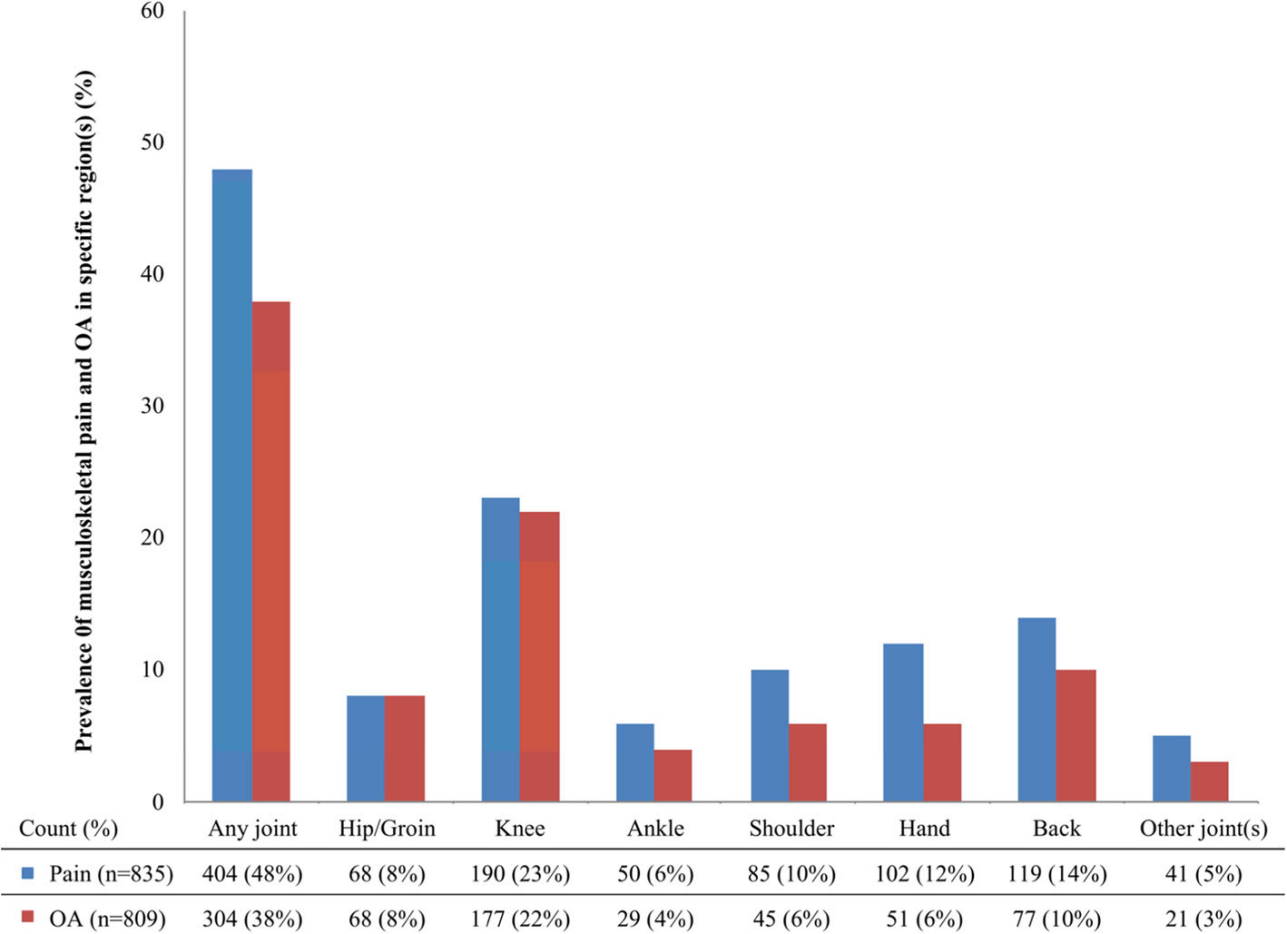
The prevalence of joint pain (on most days of the last month) and osteoarthritis (OA) in former cricketers

Joint pain on most days of the last month was reported by 48% (*n* = 404) of former cricketers, 38% (*n* = 304) had been diagnosed with OA, and 32% (*n* = 260) reported both joint pain and an OA diagnosis. Knee pain and OA were the most common (pain: 23%, OA: 22%), followed by the back (pain: 14%, OA: 10%), hand (pain: 12%, OA: 6%), shoulder (pain: 10%, OA: 6%), hip/groin (pain: 8%, OA: 8%) and ankle (pain: 6%, OA: 4%) (Fig. 2). Foot (pain: 3%, OA: 1%), elbow (pain: 1%, OA: 0%), neck (pain: 1%, OA: 1%) and wrist (pain: 1%, OA: 0%) were the most common ‘other joint(s)’ effected by pain or OA.

Crude and adjusted ORs and 95% CIs for the association between cricket-related factors and joint pain are presented in Table 2. After adjusting for cricket-related injury, bowlers had 3.1 (95% CI 1.3 to 7.4, *P* = .01) times greater odds of shoulder pain and 3.6 (1.8 to 7.4, *P* ≤ .001) times greater odds of back pain, compared to batters (Table 2). All-rounders had 1.7 (1.0 to 2.7, *P* = .04) times greater odds of knee pain and 2.1 (1.0 to 4.2, *P* = .045) times greater odds of back pain after adjusting for joint injury, compared to batters. In the crude analyses, compared with batters, wicketkeeper-batters had higher odds of shoulder and back pain, and all-rounders had higher odds of shoulder pain, however these relationships were not observed after adjusting for cricket-related injury. There was no relationship between playing position and hip/groin, ankle or hand pain (Table 2).

**Table 2:** Logistic regression investigating the association between cricket-related factors and joint pain on most days of the last month in former cricketers

Cricket-related factors		Joint pain on most days of the last month					
		Hip/Groin	Knee	Ankle	Shoulder	Hand	Back
Playing position							
Bowlers *n* = 179	Count (%)	*n* = 15 (8%)	n = 40 (22%)	n = 12 (7%)	n = 23 (13%)	n = 18 (10%)	*n* = 38 (21%)
	Crude OR	1.7 (0.7, 3.9)	1.4 (0.8, 2.3)	1.3 (0.5, 3.2)	3.5 (1.5, 8.4) **	0.9 (0.4, 1.7)	4.0 (2.0, 8.1) ***
	Adjusted OR^	1.5 (0.7, 3.7)	1.2 (0.7, 2.1)	1.2 (0.5, 3.1)	3.1 (1.3, 7.4) *	0.9 (0.4, 1.7)	3.6 (1.8, 7.4) ***
Wicketkeeper-batters *n* = 120	Count (%)	n = 8 (7%)	*n* = 25 (21%)	n = 4 (3%)	n = 13 (11%)	*n* = 19 (16%)	n = 16 (13%)
	Crude OR	1.3 (0.5, 3.5)	1.3 (0.7, 2.3)	n ≤ 5^**#**^	2.9 (1.1, 7.5) *	1.4 (0.7, 2.8)	2.3 (1.0, 5.1) *
	Adjusted OR^	1.3 (0.5, 3.5)	1.2 (0.7, 2.2)		2.6 (1.0, 6.7)	1.4 (0.7, 2.8)	2.2 (1.0, 5.1)
All-rounders *n* = 302	Count (%)	n = 30 (10%)	*n* = 82 (27%)	*n* = 22 (7%)	*n* = 33 (11%)	n = 40 (13%)	*n* = 42 (14%)
	Crude OR	2.0 (0.9, 4.4)	1.8 (1.1, 2.9) *	1.4 (0.6, 3.2)	2.9 (1.3, 6.8) *	1.2 (0.7, 2.1)	2.4 (1.2, 4.8) *
	Adjusted OR^	1.9 (0.9, 4.2)	1.7 (1.0, 2.7) *	1.5 (0.7, 3.3)	2.3 (1.0, 5.5)	1.2 (0.6, 2.1)	2.1 (1.0, 4.2) *
Batters *n* = 174	Count (%)	*n* = 9 (5%)	n = 30 (17%)	n = 9 (5%)	*n* = 7 (4%)	*n* = 20 (11%)	*n* = 11 (6%)
	Reference group						
Playing standard							
Elite standard n = 311	Count (%)	n = 31 (10%)	*n* = 75 (24%)	n = 19 (6%)	n = 30 (10%)	*n* = 49 (16%)	*n* = 52 (17%)
	Crude OR	1.4 (0.8, 2.3)	1.1 (0.8, 1.6)	1.1 (0.6, 2.0)	0.9 (0.6, 1.5)	1.8 (1.1, 2.7) **	1.5 (1.0, 2.2)
	Adjusted OR^	1.3 (0.8, 2.1)	1.0 (0.7, 1.4)	1.0 (0.6, 1.9)	0.8 (0.5, 1.3)	1.6 (1.0, 2.5) *	1.3 (0.8, 2.0)
Recreational standard *n* = 498	Count (%)	*n* = 37 (7%)	*n* = 109 (22%)	*n* = 28 (6%)	n = 52 (10%)	*n* = 48 (10%)	*n* = 60 (12%)
	Reference group						
Length of play (4-season intervals)							
	Crude OR	1.1 (1.0, 1.2)	1.0 (1.0, 1.1)	1.0 (0.9, 1.1)	1.0 (0.9, 1.1)	1.0 (1.0, 1.1)	1.0 (0.9, 1.1)
	Adjusted OR^	1.0 (0.9, 1.1)	1.0 (1.0, 1.1)	1.0 (0.9, 1.1)	1.0 (0.9, 1.1)	1.0 (0.9, 1.0)	1.0 (0.9, 1.0)

* *P <* .05, ** *P < .*01, *** *P < .*001 (highlighted in bold);

^**#**^ This analysis was not performed due to the small number of wicketkeeper-batters that reported ankle pain; ^ ‘Playing position’ and ‘playing standard’ were adjusted for region-specific cricket-related injury; ‘Length of play’ was adjusted for age and region-specific cricket-related injury;

• All results are ORs (odds ratios) and 95% CIs (95% confidence intervals);

• ‘Joint pain’ was evaluated using the following question: ‘*Have you had pain in your hip/groin knee, ankle, shoulder, hand/finger, spine/back, other joint on most days of the last month?*’;

• ‘Cricket-related injury’ was evaluated using the following question: ‘*Have you ever had any cricket-related injuries leading to more than 4 weeks of reduced participation in exercise, training or sport?*’ If yes, indicate where (hip/groin, knee, ankle, shoulder, hand/finger, spine/back, other joint);

• ‘Main playing position’: ‘*What is/was your predominant position(s) of play?*’ Multiple positions (‘Batter’, ‘Bowler’, ‘All-rounder’, ‘Wicketkeeper’, and ‘Don’t know’) could be selected; ‘batter’ category: only ‘Batter’ was selected; ‘bowler’ category: only ‘Bowler’ was selected; ‘all-rounder’ category: ‘All-rounder’ and/or both ‘Batter’ and ‘Bowler’ were selected; ‘wicketkeeper batter’ category: only ‘Wicketkeeper’ or both ‘Wicketkeeper’ and ‘Batter’ were selected; participants selecting other combinations of playing positions (e.g. ‘Wicketkeeper’ and ‘Bowler’) (*n* = 34) were excluded;

• ‘Playing standard’: ‘*What was the highest standard of cricket that you played for at least one season?*’ (‘International’, ‘County/ Premier league’, ‘Academy or county age group’, ‘University’, ‘School’, ‘Village or social’, or ‘Don’t know’). The responses were re-coded by dichotomising into ‘recreational standard’ (coded as ‘0’: university, school, village or social) vs. ‘elite standard’ (coded as ‘1’: international or county/premier league, academy or county age group). ‘Don’t know’ responses were excluded;

• ‘Length of play’: ‘*Approximately how many seasons have you played cricket for?*’ The total seasons played were re-coded into 4-season intervals (i.e. ‘1’ = 1–4 seasons, ‘2’ = 5–8 seasons, ‘10’ = 37–40 seasons etc.) ‘Don’t know’ responses were excluded.

People who had played cricket at a higher standard had 1.6 (1.0 to 2.5, *P* = .03) times greater odds of having hand pain (after adjusting for hand injury), compared with people who had only played cricket at a lower standard. Playing standard was not related to pain at other sites (Table 2). The length that participants had played cricket for was not associated with joint pain (Table 2).

## Discussion

One in every two former cricketers reported joint pain on most days of the last month and 38% had been diagnosed with OA. The most common sites for joint pain were the knee, back, hand and shoulder, and OA was most prevalent in the knee, spine and hip. After adjusting for injury, bowlers had greater odds of shoulder and back pain, and all-rounders had greater odds of knee and back pain, compared to batters. Elite cricketers had greater odds of hand pain compared to recreational cricketers, however playing elite cricket was not associated with increased odds of pain at other sites. There was also no association between length of play and joint pain.

In this sample of former recreational and elite cricketers aged 30–93 years, 38% of individuals had been diagnosed with OA and 48% had persistent joint pain. In comparison, the English Longitudinal Study of Ageing reported an OA prevalence of 13% in a general population sample aged ≥50 years [9]. Additionally, the Consultations in Primary Care Archive in England found that one-in-five individuals aged 45 years and over in North Staffordshire, England consult a General Practitioner for joint pain or OA, annually [21]. These figures suggest that joint pain and OA are more prevalent in former cricketers than in the general population. A high prevalence of OA has also been reported in former contact and collision sport athletes; 49% of former professional soccer players reported a diagnosis of OA at an average age of 40 ± 13 years [22]; 36% of retired American Football (National Football League) players aged 24–95 years self-reported experiencing OA [23]; and as many as 60% of former rugby-union players aged ≥50 years had been diagnosed with OA [24]. The high joint pain and OA prevalence in former sport participants highlights the importance of developing and implementing strategies to prevent OA and optimise long-term joint health in this population. Primary prevention of knee OA through injury prevention strategies, has received great attention in recent literature and many effective strategies have been trialled that reduce knee injury prevalence in cutting, pivoting and collision sports [25–27]. However, in former cricketers, pain and OA also effected other joints, including the spine, hand and shoulder. Very little research exists investigating the efficacy of injury prevention strategies targeting these joints.

Former bowlers had greater odds of back and shoulder pain, and former all-rounders had greater odds of back and knee pain, compared to former batters, and these relationships remained after accounting for joint injuries. The greater odds of joint pain in bowlers and all-rounders may be due to increased workload and forces in training and competition, combined with the biomechanical demands of bowling [28–30]. Bowlers usually perform greater competition distance and sprinting than other playing positions, increasing overall training load compared to other positions [28]. Further, during bowling, the excessive amounts of lateral trunk flexion, front-foot contact with an extended lower limb, and shoulder counter-rotation can predispose bowlers to injury and joint pain [31–34]. More specifically, a cricketer’s spine is typically in a laterally flexed, rotated and hyperextended position at bowling release, when ground reaction forces are at their greatest [8, 35]. During the bowling front foot strike, cricketers can be subject to a peak vertical force of 3.8 to 9.0 times body weight [8, 36, 37]. This excessive ground reaction force may predispose cricketers to lower back and lower-limb joint pain. Additionally, bowling requires substantial repetitive shoulder rotational motion, resulting in high shoulder loads [38, 39]. Strategies to prevent injury in cricketers (including upper extremity and lower-limb strengthening, trunk extensor endurance, and neuromuscular control exercises [34, 40–42]) and interventions to improve bowling biomechanics and technique, may have important implications for long-term musculoskeletal health.

Playing cricket at a higher standard was associated with a greater prevalence of hand pain. Notably, this relationship remained after adjusting for hand injuries that resulted in more than 4 weeks of reduced exercise participation. It is possible that sustaining multiple minor hand injuries, that result in < 4 weeks of exercise restriction, predispose a cricketer to hand pain in later life. Elite cricketers have been shown to bowl at significantly greater velocities compared to sub-elite cricketers [43]. The increased bowling velocity results in the ball contacting the hands of batters and wicket-keepers with greater force, and elite batters are also likely to translate this into greater ball speed off the bat and therefore higher forces in fielding. It is possible that the repetitive loads and forces that an elite cricketer’s hands are exposed to throughout their playing career, contributed to the high rate of hand pain observed in our study. However, these explanations are speculative, highlighting the need for further research investigating risk factors for hand pain after retirement from cricket.

Interestingly, despite a higher incidence of injury amongst elite cricketers (compared to recreational cricketers) [44], there was no difference in the prevalence of pain at other joints. Elite cricketers, although exposed to more injury, may have had better access to high-quality medical care enabling an earlier and more accurate diagnosis and optimal injury management and rehabilitation. Furthermore, elite cricketers would have had more time and resources to engage in injury rehabilitation compared to recreational sport participants who would likely have other work and time commitments. Former elite cricketers may also possess distinct psychological characteristics, including mental toughness, resilience and enhanced pain coping strategies [45, 46]. Such psychological traits could result in less elite cricketers reporting that they experience joint pain on most days of the last month, compared with recreational cricketers.

There was no relationship between length of play and joint pain in former cricketers. A potential explanation for this is opposing relationships, resulting in no observed effect. For example, a cricketer who suffers joint pain may be more likely to stop playing cricket earlier than someone who is pain-free (37% of participants stopped playing cricket due to chronic pain or injury). On the other hand, someone who plays cricket for a greater number of seasons may have an increased risk of joint injury and increased exposure to repetitive joint loading, which could increase odds of joint pain after cricket retirement. Additionally, the participants had played cricket for median 33(IQR 21–41) cricket seasons, it is possible that the average cricket-seasons played was too high to observe an effect. In the analysis, we did not account for the various reasons for retirement from cricket or other sports played throughout a participant’s lifetime; further research exploring this may provide additional insights regarding the relationship between playing duration and joint pain in former cricketers.

A strength of this study was the large sample size, providing sufficient power to investigate pain in joints that have little or no research in former cricketers (including hand, ankle and hip). It was also novel to include a substantial proportion of recreational cricketers in this study; research in this group of cricketers is rare despite a majority of cricket participants playing at a recreational level. There is an inherent potential for recall bias with regards to participants recollecting their cricket-related injury history and other cricket related information [47]. To minimise likelihood for recall bias we included ‘don’t know’ response options for all items requiring recollection. There are also limitations regarding the method for assessing OA in this sample. The gold standard would involve clinical assessment of OA symptoms for all participants [5]. However, due to the study design and recruitment strategy, this was not feasible. It is likely that a proportion of former cricketers had OA but had not received an OA diagnosis. Conversely, the discord between radiographic findings and symptomatic OA is now well established [48, 49], some participants may have incorrectly received an OA diagnosis based only on radiographic evidence. Considering these limitations, joint pain on most days of last month was considered a more clinically relevant outcome to be included in regression analyses. In addition, although cricket-related injury and age were considered the most important confounders to adjust for in analyses, there is a possibility that unmeasured confounders could have biased effect estimates. Potential unmeasured confounders include use of protective equipment and playing other sports at the time of cricket participation. We recommend that future studies account for these potential confounders by incorporating their assessment into the study design. Finally, only 9% of individuals who received an invitation responded and consented to participate in the Cricket Health and Wellbeing Study. It is possible that some people who did not respond were ineligible, or did not read the invitation email. It is also possible that those who responded to the invitation were more likely to experience joint pain or OA than the non-responders. Unfortunately, we did not have data on non-responders to compare characteristics with eligible study participants.

## Conclusions

Every second former cricketer who participated in this study experienced pain on most days of the last month, and more than one in three had been diagnosed with OA. Compared with former batters, former bowlers had greater odds of shoulder and back pain, and former all-rounders had greater odds of back and knee pain. These relationships were not explained by a higher prevalence of injury in former bowlers and all-rounders. Former elite cricketers had greater odds of hand pain compared to former recreational cricketers, however a higher playing standard was not related to increased odds of pain at other sites.

## Data Availability

Specific anonymised data from this study may be available upon reasonable request from the authors.

## Abbreviations

95% CI: 95% confidence interval
BMI: Body mass index
IQR: Interquartile range
OA: Osteoarthritis
OR: Odds ratio
QOL: Quality of life
